# Successful Encoding during Natural Reading Is Associated with Fixation-Related Potentials and Large-Scale Network Deactivation

**DOI:** 10.1523/ENEURO.0122-18.2018

**Published:** 2018-11-08

**Authors:** Naoyuki Sato, Hiroaki Mizuhara

**Affiliations:** 1 Department of Complex and Intelligent Systems, School of Systems Information Science, Future University Hakodate, Hokkaido 041-8633, Japan; 2 Graduate School of Informatics, Kyoto University, Sakyo-ku, Kyoto 606-8501, Japan

**Keywords:** memory encoding, natural reading, neural oscillation

## Abstract

Reading literature (e.g., an entire book) is an enriching experience that qualitatively differs from reading a single sentence; however, the brain dynamics of such context-dependent memory remains unclear. This study aimed to elucidate mnemonic neural dynamics during natural reading of literature by performing electroencephalogram (EEG) and functional magnetic resonance imaging (fMRI). Brain activities of human participants recruited on campus were correlated with their subsequent memory, which was quantified by semantic correlation between the read text and reports subsequently written by them based on state of the art natural language processing procedures. The results of the EEG data analysis showed a significant positive relationship between subsequent memory and fixation-related EEG. Sentence-length and paragraph-length mnemonic processes were associated with N1-P2 and P3 fixation-related potential (FRP) components and fixation-related θ-band (4–8 Hz) EEG power, respectively. In contrast, the results of fMRI analysis showed a significant negative relationship between subsequent memory and blood oxygenation level-dependent (BOLD) activation. Sentence-length and paragraph-length mnemonic processes were associated with networks of regions forming part of the salience network and the default mode network (DMN), respectively. Taken together with the EEG results, these memory-related deactivations in the salience network and the DMN were thought to reflect the reading of sentences characterized by low mnemonic load and the suppression of task-irreverent thoughts, respectively. It was suggested that the context-dependent mnemonic process during literature reading requires large-scale network deactivation, which might reflect coordination of a range of voluntary processes during reading.

## Significance Statement

Context-dependent memory encoding during natural reading of literature was evaluated using electroencephalography (EEG) and functional magnetic resonance imaging (fMRI) based on a subsequent memory paradigm. Subsequent memory was quantified by semantic correlation between the read text and reports subsequently written by the participants, based on a recent natural language processing procedure. Our results demonstrated a positive correlation between subsequent memory and fixation-related EEG and a negative correlation with fMRI activity. Sentence-length and paragraph-length processes were associated with regions belonging to the salience network and the default mode network, respectively. This is the first demonstration that memory encoding during literature reading is associated with large-scale network deactivations, which might reflect the coordination of a range of voluntary processes during reading.

## Introduction

Neural dynamics during memory encoding have been extensively investigated using the subsequent memory paradigm (Brewer, 1998; Wagner, 1998), in which brain activity during encoding of items that are subsequently remembered is compared to the activity during encoding of items that are subsequently forgotten. Functional magnetic resonance imaging (fMRI) studies have demonstrated that multiple brain regions, including the inferior frontal cortex and hippocampus, are activated during successful memory encoding of words (Wagner, 1998), pictures (Brewer, 1998), and item-context associations (Summerfield and Mangels, 2005; for review, see Kim, 2011). This activation is termed the subsequent memory effect (SME). In contrast, the activation of distinct brain regions, such as the anterior and posterior midline cortices, was also observed during unsuccessful encoding; an effect termed the “subsequently forgotten effect” or “negative subsequent memory effect” (NSME; Otten and Rugg, 2001; Wagner and Davachi, 2001; Daselaar et al., 2004).

Electroencephalography (EEG) studies found that specific event-related potentials (ERPs) and brain oscillations occur during the SME. Specifically, the P3 ERP component increases during successful encoding of words (Klimesch et al., 2000). EEG θ (4–7 Hz) oscillations increased during successful encoding of words (Klimesch et al., 1996), pictures (Osipova et al., 2006), and item-context binding (Summerfield and Mangels, 2005; for review, see Nyhus and Curran, 2010) . However, opposite effects, namely EEG θ decreases during successful encoding, have also been reported, suggesting that the SME primarily reflects perceptual and cognitive processes engaged by the encoding tasks (Hanslmayr and Staudigl, 2014). In these studies, relatively simple memory contents, such as single words or item-location pairs have been used. Thus, it is of interest to investigate whether the same neural dynamics is relevant for the encoding of a more natural form of memory consisting of semantically richer and context-dependent material, for example the experience of reading literature.

Literature reading provides a good example of a semantically rich and context-dependent experience for evaluating natural memory. The neural dynamics of reading has been extensively tested with sequential presentation of words constituting sentences, using EEG (Kutas and Hillyard, 1980; Kutas and Federmeier, 2000; Hagoort et al., 2004; Bastiaansen and Hagoort, 2006) and fMRI (Cutting et al., 2006; Pallier et al., 2011). Moreover, the neural dynamics of narrative-level context have been evaluated by EEG and fMRI studies (Ferstl and von Cramon, 2001; Xu et al., 2005; Hasson et al., 2007; Yarkoni et al., 2008; Brennan, 2016). Some of these studies (Hasson et al., 2007; Yarkoni et al., 2008) demonstrated a subsequent memory effect during narrative comprehension. However, the examination of semantic contents after reading in these studies was based on a small number of questions regarding the text, and these were found to be too abstract for capturing the entire semantics of the text being read. Thus, the mnemonic process relevant to semantically rich content during reading needs further elucidation.

Recently developed natural-language techniques are expected to be useful for quantifying the semantic content of subsequent memory. One of these is “distributed semantic representation,” in which each word is transformed to a vector consisting of intermediate semantic features. This technique is used to perform text comparisons based on semantic correlation rather than the appearance of particular keywords (Blei et al., 2003; Mikolov et al., 2013). The same technique has already been employed in fMRI studies showing that intermediate features were associated with widely distributed cortical regions (Mitchell et al., 2008; Huth et al., 2012). These studies are important in that they support the plausibility of using intermediate features in the investigation of brain activities related to semantics. Given the assumption that subsequent memory performance depends on a particular pattern of cortical activation during encoding into long-term memory, these same intermediate features may be used for the evaluation of subsequent memory of the texts.

In this study, we aimed to elucidate mnemonic neural dynamics during natural reading of literature using EEG and fMRI measurements. Brain activity was analyzed by comparing the measurements with content reports subsequently written by the participants based on a recently developed natural language technique quantifying semantic correlations to body of text. EEG measurement was combined with eye tracking to enable FRP analysis during reading, by which neural dynamics during free-viewing was assessed, while solving the problem of ocular artifact contamination in EEG signals (Dimigen et al., 2011; Henderson et al., 2013). We specifically asked the following questions: (1) What neural dynamics underlies memory encoding during natural reading of literature? (2) What neural dynamics is associated with multi-scaled contextual processing during reading of sentences and paragraphs?

## Materials and Methods

We performed EEG and fMRI measurements separately during natural reading of identical texts. The task procedures and statistical analyses in these two experiments were designed to be comparable.

### EEG methods

#### Subjects

Fifteen volunteers (two female, two left-handed, native Japanese speakers; 20–34 years old; mean ± SD: 22.5 ± 3.5 years old) were recruited via poster advertisement at Future University Hakodate. They had no experience of neurologic disorders or use of psychotropic medications and had normal visual acuity, except that the eyes of six participants were corrected by spectacles and the eyes of one participant were corrected by intraocular lenses. They were compensated $25–30 for participating in the study; the exact amount was prescribed by the city and depended on the school year of the participant in the Future University Hakodate. They provided written informed consent before participating in the experiment. The study was approved by the Ethics Committee of the Future University Hakodate. Data from the two left handed volunteers were ultimately excluded from the analysis.

#### Stimuli

Four scientific essays written by Torahiko Terada, entitled “Eagle’s eye and olfaction,” “Rhythms of poems,” “Physiologic responses to seeing a movie,” and “A case study of a ghost,” were used as stimuli (Aozora Bunko, http://www.aozora.gr.jp/index_pages/person42.html). They were selected as logical and non-emotional texts, suitable for high school students (unlike scientific articles). Each text was modified to modern kana (Japanese syllabary spelling system) with a length of 1919.5 ± 77.6 words (2973.3 ± 137.3 characters, sentence length: 46.2 ± 22.9 words, paragraph length: 116.2 ± 82.4 words; mean ± SD). Their readability was measured as having a grade of “beyond high school (13)” using a program based on a statistical language model, Obi-2 (Sato et al., 2008).

During reading, the essays were presented on a 21-inch monitor (Sony, CPD-G520), as a line of segmented text with 40 characters, displayed as 985 × 24 pixels (subtending 27.9 × 0.74°) in white, on a black background. The single line presentation was used to restrict eye movements to horizontal saccades or eye blinks (Dimigen et al., 2011). The participants voluntarily advanced to the next line by pushing a button with the right thumb. Returning to a previous line was not allowed.

#### Procedure

Following the placement of EEG electrodes on the participants’ heads, they read the four essays in a random order with rest intervals. During reading, the head position was stabilized by chin and forehead rests. At the beginning of each reading session, the eye movement system was calibrated with a nine-point grid. Following the reading session, the electrodes were removed, and the subjects washed their hair to remove conducting gel (which lasted for ∼15 min). The subjects were then seated at a desk and wrote a summary of the content read (content report), as detailed as possible, following the order in which the essays were read. During this procedure, the interval between essay encoding and retrieval was ∼30 min, and no explicit opportunities for rehearsing the essays were included. Possible influence of rehearsals during the interval was thought to be reduced by the separation of particular encoding and retrieval pairs using other essays tasks and the counter-balanced order of the text presentation. Before the main experiment, subjects performed a training session with a short essay (714 words) to familiarize themselves with the task.

#### Eye movement data acquisition

Eye movements were recorded binocularly with an infrared video-based eye tracker (EyeLink CL, SR Research Systems) at a sampling rate of 250 Hz. During reading, saccades were detected by EyeLink software using an eye-movement velocity threshold of 30°/s, an acceleration threshold of 8000°/s^2^ and a saccadic threshold of 0.15°. Data from the right eye were analyzed, and those from the left eye were used only for validation. The following atypical fixations were discarded from the analysis: fixations separated vertically by 48 pixels (two characters) from the line, fixations with a duration of <50 ms or >750 ms, fixations at the first or the last saccades during the reading of each line, fixations for small (<1 character) or large (>20 character) saccades, and fixations shortly preceding/following an eye blink (ranging from -500 to 1000 ms of blink onset).

#### EEG data acquisition and preprocessing

EEG and EOG data were acquired using Ag/AgCl electrodes with a BrainVision amplifier (BrainProducts). Twenty-one electrodes were mounted on the scalp according to the standard 10–20 system without Fp1 and Fp2. Four EOG electrodes were affixed to the left and right outer eye canthi and above and below the right eye. EEG data (0.01- to 100-Hz bandpass, 500 Hz sampling rate, impedance of the electrode 12.6 ± 11.6 kΩ; mean ± SD) were referenced to the FCz electrode during measurements and re-referenced to the average signal recorded at electrodes placed on the two earlobes for analysis.

Ocular artifacts were corrected by independent component analysis (Henderson et al., 2013). First, a dataset dominantly including ocular artifacts, given by fixation-related data from -120 to 50 ms from the fixation onset [20,443 ± 11,008 time points (481.8 ± 259.0 trials; mean ± SD) × 26 electrodes, with additional bandpass filtering between 1 and 50 Hz], were collected and their independent components were calculated by FastICA (Hyvärinen and Oja, 2000). Second, independent components highly correlated to either horizontal, vertical, or radial EOGs (correlation coefficient of the entire time course across trials > 0.15) were discarded, and the same separation matrix, calculated from the subset of the data, was applied to the original data. By this procedure, 5.5 ± 2.3 components (mean ± SD) were discarded; this rejection rate was similar to the rate reported previously (Henderson et al., 2013). Finally, the corrected EEG signals were filtered between 1 and 40 Hz (using a zero-lag Butterworth filter with -12 dB/octave roll-off) and down-sampled to 250 Hz to match the eye-movement data. EEG and eye movement data were synchronized by a common trigger input from the stimulus computer.

#### Text data analysis

**Figure 1.**
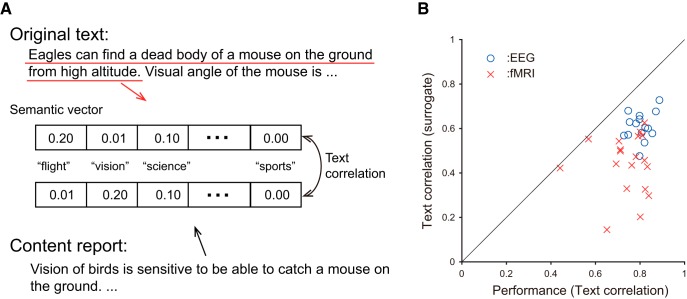
Computation of text correlations between the text that was read and the content report subsequently written by the participants. ***A***, Schematic procedure for text comparison. Each text and content report were translated into a semantic vector consisting of intermediate semantic features computed from word-collocation in a large text database. The correlation of the vectors represents the semantic similarity between the two texts rather than the appearance of specific keywords (see text for details). ***B***, Performance of EEG and fMRI participants. Blue and red plots indicate the participants in the EEG and fMRI experiments, respectively. Horizontal and vertical axes denote the performance (the entire text correlation between the text read and the content report) and the surrogate text correlation, respectively. The fact that plots lie below the diagonal line shows that the content reports specifically reflected the texts read. The data from two participants, for whom the plots appeared along the diagonal line and whose correlations were below 0.6, were excluded from the analysis.

The analysis of the text data can be outlined as follows (Fig. 1*A*). Each text consisting of an arbitrary number of words was translated into a semantic vector consisting of intermediate semantic features (Blei et al., 2003; Mikolov et al., 2013). Statistically, natural texts consist of approximately ten thousand types of words and the collocation matrix appears sparse. Therefore the relationship between the words (i.e., collocation matrix) can be computationally compressed and the dimension of the compressed word relationship typically falls within the range of several hundred dimensions (Landauer and Dumais, 1997). This compressed word relationship produces a word-to-vector map. When a text is represented by the average of the word vectors relevant to the words in the text (“bag-of-words”), the averaged vector, termed the “semantic vector,” captures the intermediate semantics of the text, in a way that is robust against the influence of synonyms or rephrasing. For example, when two intermediate semantic features represent the amount of “flying” or “vision” (Fig. 1*A*), a semantic vector represents its semantics by the combined amounts of these semantic features, although in reality these semantic features were automatically produced by the above algorithms to optimally cover natural texts. The text that was read and the content reports subsequently written by the participants were individually translated into semantic vectors, and their correlation was used as an index for semantic text correlation evaluating the subsequent memory.

The detailed procedure applied in the text data analysis consisted of the following steps.

##### Step 1

The vector features of all words were computed from word-occurrence data within a large text corpus [Balanced Corpus of Contemporary Written Japanese (BCCWJ; Maekawa et al., 2014); Library/Book sub-corpus (10,551 texts, 60,615 word types, fixed length of 1000 characters per text) using the algorithm Word2Vec (Mikolov et al., 2013); using the parameters: context window of 10 words, CBOW model, and negative word sampling of 15 words)]. For this process we used open source code from https://code.google.com/archive/p/word2vec/.

##### Step 2

The words in every text were segmented into morphologic units (“short-unit word”) using the Japanese morphologic analyzer MeCab (http://taku910.github.io/mecab/) with the dictionary unidic-mecab (ver.2.1.3; https://ja.osdn.net/projects/unidic/).

##### Step 3

Each open-class word (noun, verb, adverb, and adjective) within the text was represented by a 100-dimensional vector $w_{i}$, where i denotes word position in the given text.

##### Step 4

Three types of text correlation were calculated using sentence-length samples, paragraph-length samples, or the entire text. In the calculation, each text unit was represented by the average of the word vector, $T_{\text{T}}=\frac{1}{\left|\text{T}\right|}\sum_{i\in \text{T}}w_{i}$, where $w_{i}$ is the i-th word vector in the text, and T is the set of word IDs in the text. The correlation of a pair of texts represented by the semantic vectors $T_{\text{E}}$ and $T_{\text{R}}$ was determined by cosine similarity, $C(T_{\text{E}},T_{\text{R}})=T_{\text{E}}T_{\text{R}}/(|T_{\text{E}}||T_{\text{R}}|)$, where the values range from -1-1, 0 indicates an independent text pair, and 1 indicates an identical text pair.

##### Step 5

The correlation between the entire text read and the content report was termed the “performance” and used as a quality index for the individual content reports. To clarify whether the content reports specifically reflected the corresponding texts, surrogate text correlations, calculated as the average of correlations between the content report and non-corresponding texts, were additionally computed. The feature dimension (100) was determined to maximize the difference between the text read-content report correlation and the surrogate text correlations.

##### Step 6

In the following subsequent memory analysis, each sentence or paragraph of the text read was compared with the entire content report to identify which part of the text was reflected in the content report. The sentence-length correlations changed quickly as a function of word position, and paragraph-length correlations slowly because the former reflected the appearance of specific sentences while the latter reflected the appearance of abstract themes governing several sentences forming a paragraph. In the current analysis, text correlation was not highly sensitive to variation of word length in sentences or paragraph (i.e., results with fixed-length text correlations using the averaged sentence- or paragraph-length were fundamentally unchanged from the current results; data were partially shown by the author (Sato, 2015), thus the influence was not corrected.

### EEG data analysis

The corrected EEG signals were analyzed in terms of FRPs and fixation-related time-frequency power as follows. First, the corrected EEG signals were segmented from -500 to 1000 ms from the fixation onset given by the eye movement data. Segments including data points within a limit of ±80 μV were used. Second, the influence of overlapped FRPs from neighboring fixations was reduced by subtracting an estimated FRP calculated from de-convolution by the use of the adjacent response technique (Woldorff, 1993). Third, fixation-related time-frequency EEG power was calculated using complex Morlet wavelet transformation (width = 5), where 19 frequency bands were determined on a logarithmic scale (2 ^ (1, 1.25, …, 5.5) Hz) and split into four distinct bands, 4–7 Hz (θ), 8–12 Hz (α), 14–28 Hz (β), and 30–48 Hz (γ). The baseline for each power was subtracted; the baseline was calculated from the period within -300 to -100 ms from the fixation onset. Finally, the time-frequency EEG power at time t and frequency f, P(t,f), was compared with the text correlation between the part of the text read at eye fixation and the content report. Two text correlations, one at sentence-length and another at paragraph-length, $C_{S}$ and $C_{P}$, were used in the analysis. The time-frequency power was analyzed by multiple regression using the two text correlations, as (Eqn. 1):

(1) $$P(t,f)=b_{0}+b_{S}C_{S}^{\prime }+b_{P}C_{P}^{\prime },$$

where $C_{S}^{\prime }$ and $C_{P}^{\prime }$ are the sentence-length and paragraph-length text correlations with a modification of Gram-Schmidt orthogonalization. The regression coefficients $b_{S}$ and $b_{P}$ were calculated separately for each electrode, each time point and each frequency band, and then integrated across all subjects using the t statistic. Multiple comparisons in the t tests were corrected using the nonparametric clustering permutation test (Maris and Oostenveld, 2007) with 4000 shuffled data sets, in which the statistical threshold was provided by a single procedure taking into account the electrodes, time, and frequency simultaneously. FRPs were also analyzed using the identical regression analysis, where the regression coefficients were calculated separately for each electrode and each time point and then integrated across all subjects using the t statistic. Additionally, to quantify the potential contribution of ocular artifact residuals in the corrected EEG, saccade size and fixation duration were analyzed using the same type of regression analysis.

### fMRI methods

#### Subjects

Nineteen volunteers (10 female, all right-handed, native Japanese speakers; aged from 21 to 29 years old; mean ± SD: 23.9 ± 2.5 years old) were recruited via poster advertisement at Kyoto University. They had no experience of neurologic disorders or use of psychotropic medications and had normal visual acuity, except that the eyes of 13 participants were corrected by spectacles. They were compensated $65 for participating in the study. Written informed consent was provided before participating in the experiment. The experimental protocol was approved by the ethics committee at the Unit for Advanced Studies of the Human Mind, Graduate School of Medicine and the Faculty of Medicine, Kyoto University. Data from two volunteers were excluded from the analysis because of low memory performance.

#### Procedure

The procedure was as described above for the EEG experiment. The participants read the two essays at a natural pace within an MR scanner for 10 min. The texts were two out of four essays used in the EEG experiment, the titles of which were “Eagle’s eye and olfaction” and “Physiologic responses to seeing a movie.” While reading, two lines of the text (60 characters) were displayed, with the participant voluntarily advancing to the next page by pressing a button (the essays consisted of 50 and 54 pages, respectively). To have page transition intervals longer than the timescale of hemodynamic response (for stable regressing out of the influence of page transition and button pressing in the following analysis), the number of characters in a page was changed to be larger than that of the EEG experiment. After the two texts were read, a 5-min structural scan was performed. Following structural scanning, the participants sat at a desk outside the scanner and wrote two content reports within 15 min, following the order in which the essays were read in as much detail as possible. In this procedure, the interval between essay encoding and essay retrieval was ∼20 min.

#### fMRI data acquisition and preprocessing

During reading, blood oxygenation-sensitive echoplanar images (EPIs) were acquired using the 3T MR scanner (Magnetom Verio, Siemens) under the following conditions: repetition time = 2 s, echo time = 30 ms, flip angle = 80°, field of view = 192 mm, in-plane resolution = 64 × 64, 30 axial slices, slice thickness = 5 mm. One session lasted for ≤10 min (297 scans) as defined by the reading time for each essay. Two sessions were performed, one for each essay. After reading, a T1-weighted anatomic volume was acquired.

We used SPM8 software (Wellcome Department of Cognitive Neurology, London, United Kingdom; www.fil.ion.ucl.ac.uk/spm) for image preprocessing and voxel-based statistical analysis. The initial five scans in each session were discarded from the analysis to eliminate magnetic saturation effects. The remaining EPIs (≤292 scans × two sessions) were mapped to the first image volume for each participant to correct for head motion. The slice timing was corrected with respect to the middle slice to remove the time delay of scanning the entire brain. The individual EPIs were normalized to a standard brain by applying the parameters estimated by matching the T1 anatomic image to the stereotactic image in Montreal Neurologic Institute coordinates. The EPIs were then smoothed with an 8-mm full-width at half-maximum Gaussian kernel.

### fMRI analysis

A voxel-based statistical analysis was performed on the preprocessed EPIs. The blood oxygenation level-dependent (BOLD) responses were evaluated using a general linear model including regressors of interest, which were the sentence-length and paragraph-length text correlations between the text read and the content report. The text correlations were calculated identically to those in the EEG analysis, except for one parameter in Word2Vec (a context window parameter was changed from 10 to 15 words to maximize the difference between the correlations of the content report with the text read and the correlations with non-corresponding texts). In contrast to the fixation-related EEG analysis, the text correlation was associated with the BOLD signals at each scanning time, in which many eye fixations were included. To identify neural mechanisms underlying text correlations, we hypothesized an expected BOLD response by convolving the canonical hemodynamic response function with the text correlations for each participant and session. The model additionally included the time taken to press the button and six motion regressors obtained from the registration process.

Before performing the regression analysis, low-frequency confounding effects were removed using a high-pass filter with a 120-s cutoff period, and serial correlations among the scans were estimated using an autoregressive model [AR(1)] to remove the high-frequency noise contaminating the EPI time series. The parameter estimates were computed for each subject using a fixed-effects model and then taken into the group analysis using a random-effects model of a *t* statistic (uncorrected *p* < 0.001, cluster-wise FDR; *p* < 0.05).

## Results

### Behavioral results

The 13 participants who underwent EEG measurements took a mean time of 7.4 min (SD, 2.4 min) to read each of four essays and write four content reports with a mean length of 205.8 words (SD, 130.8 words) in a mean time of 12.0 min (SD, 6.4 min). The performance (the entire text correlation between the content reports and the text read) was calculated as 0.80 ± 0.05 (mean ± SD) which was significantly larger than the surrogate text correlation (0.61 ± 0.06; paired *t* test, *t*_(12)_ = 102.7, *p* < 0.001). It was therefore clearly demonstrated that the participants properly described the content of the text read (Fig. 1*B*).

In the fMRI experiment, all 17 participants read each of the two essays during a mean period of 8.2 min (SD, 1.9 min; 246.4 ± 55.8 volumes) and wrote content reports having a mean length of 281.3 words (SD, 85.5 words) within 10 min. The memory performance was calculated as 0.77 ± 0.06 (mean ± SD; *t* statistic comparing the text correlation to surrogate text correlation (0.44 ± 0.13); *t*_(16)_ = 52.5, *p* < 0.001). The difference between the memory performance of fMRI participants and that of EEG participants was not significant (*t*_(28)_ = 1.74, *p* = 0.09), suggesting that cognitive processes in the fMRI versus EEG participants were comparable.

### EEG results

After rejecting atypical saccades (defined by atypical fixation duration or saccade size or too small separation from blinks), a mean of 481.8 fixations (SD, 259.0 fixations) was analyzed for each text and each participant. The mean duration of fixation was 287.5 ms (SD, 92.7 ms), and the mean saccade size was 2.1 characters (SD, 4.7 characters). These values agreed with the typical values recorded during reading in English (Rayner, 1998).

**Figure 2.**
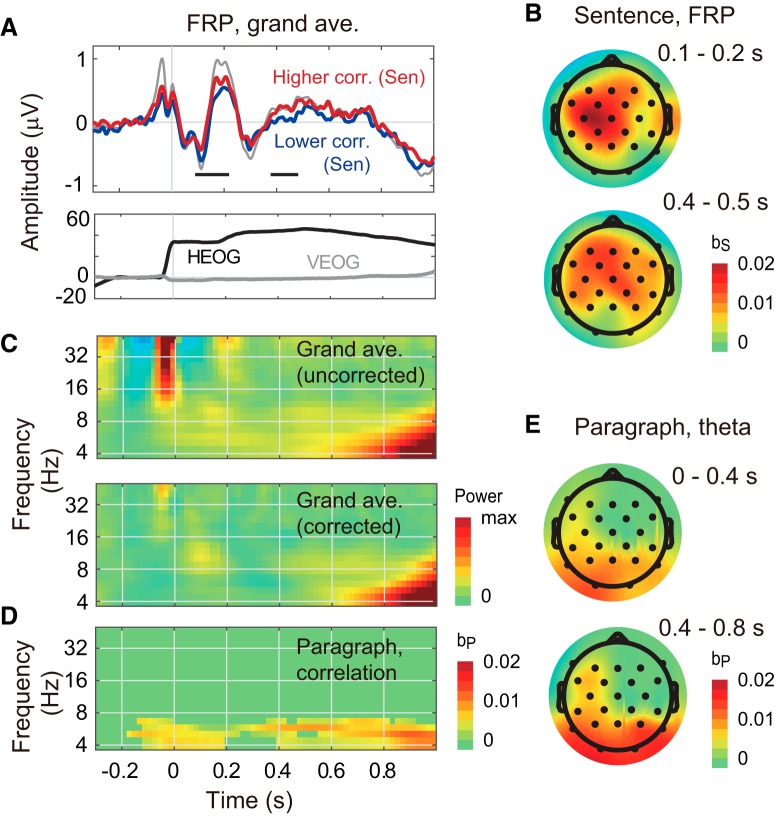
Results of fixation-related EEG analysis. ***A***, FRPs of grand averaged signal over all electrodes. The upper plot shows the FRPs. The red and blue plots show FRPs during higher and lower text correlation between the read text and content reports, respectively. The “higher” and “lower” regression coefficients were defined by a median split of the regression coefficients. The gray plots show uncorrected FRP. Horizontal bars indicate time points showing significant effects (*p* < 0.05). Multiple comparisons were corrected by the nonparametric clustering permutation test (Maris and Oostenveld, 2007). The horizontal axis represents the time from fixation onset. The lower plot shows horizontal and vertical EOGs (HEOG and VEOG). ***B***, Topographical head maps of regression coefficients of the sentence-length text correlation in the time periods 0.1–0.2 and 0.4–0.5 s. ***C***, Time-frequency maps of raw (ocular-artifact-uncorrected) and corrected EEG power. The horizontal axis represents the time from fixation onset; vertical axis indicates frequency on a logarithmic scale. ***D***, Time-frequency map of regression coefficients averaged over all channels for the paragraph-length text correlations between the text read and the content report. The values were masked by a cluster-based statistical value (*p* < 0.05). ***E***, Topographical head maps of regression coefficients of the sentence-length and paragraph-length text correlations in θ-band (4–8 Hz) EEG. The maps of regression coefficients were averaged over the time periods 0–0.4 and 0.4–0.8 s.

Figure 2*A* shows FRPs of the grand averaged signal. The red and blue plots show FRPs related to higher and lower regression coefficients for the sentence-length comparison, where higher and lower regression coefficients were defined by a median split of the regression coefficients. The shape of the FRPs appeared similarly to those reported during reading by Dimigen et al. (2011). In the FRP analysis, there were 65 clusters of (electrode, time)-samples (60 positive and five negative) showing the sentence-length subsequent memory effect and 45 clusters of samples (19 positive and 26 negative) showing paragraph-length effects. The two positive clusters showing a sentence-length effect, in the time periods 0.10–0.21 s (*p* = 0.018) and 0.38–0.48 s (*p* = 0.025), had Monte Carlo *p* values that were <0.025 (Fig. 2*A*). As also shown in Figure 2*B*, both clusters broadly distributed over the scalp, while the former and the latter appeared in the left central area and in the frontal area, respectively. The former and latter clusters appeared to be associated with N1-P2 complex and P3 components, respectively. There were no significant clusters showing a paragraph-length effect. Topographic maps of the resulting regression coefficients averaged over time periods of either 0.1–0.2 or 0.4–0.5 s from fixation onsets (Fig. 2*B*) showed that the sentence-length effect appeared from the frontal to central regions.

Figure 2*C* shows fixation-related time-frequency maps for the raw (ocular-artifact uncorrected) and corrected EEG powers averaged over all electrodes. In the corrected time-frequency map, the power at the fixation onset, which is thought to reflect ocular artifact, was greatly reduced. However, the power in the low frequency band in the period of >0.8 s, which was influenced by eye blinks at >1 s, remained uncorrected. In the analysis of fixation-related time-frequency EEG power, there were 344 clusters of (electrode, time, frequency)-samples (187 positive and 137 negative) in the sentence-length comparison and 317 clusters of samples (162 positive and 155 negative) in the paragraph-length comparison. Only one positive cluster, for the θ-band in the paragraph-length comparison (*p* = 0.038), had a Monte Carlo *p* value that was <0.025. There were no significant clusters in the sentence-length comparison. Figure 2*D* shows the fixation-related time-frequency map for regression coefficients averaged over all electrodes in the paragraph-length comparison. The topographic maps of the resulting regression coefficients of θ-band EEG power averaged over time periods of either 0.0–0.4 or 0.4–0.8 s from fixation onsets (Fig. 2*E*) showed that the paragraph-length effect appeared over the occipital region and left fronto-temporal region.

No eye movement-related parameters showed a significant correlation to subsequent memory in sentence-length or in paragraph-length comparisons (saccade size; sentence-length correlation, *t*_(12)_ = 1.15, *p* = 0.27, paragraph-length correlation, *t*_(12)_ = 0.47, *p* = 0.64, fixation duration; sentence-length correlation, *t*_(12)_ = -1.93, *p* = 0.08; paragraph-length correlation, *t*_(12)_ = 1.96, *p* = 0.07). This result suggests that the significant correlation between fixation-related EEG and subsequent memory did not result from contamination by ocular artifacts. An additional analysis using three regressors (sentence-length and paragraph-length text correlations and fixation duration) yielded results that were almost identical (data not shown).

### fMRI results

**Figure 3.**
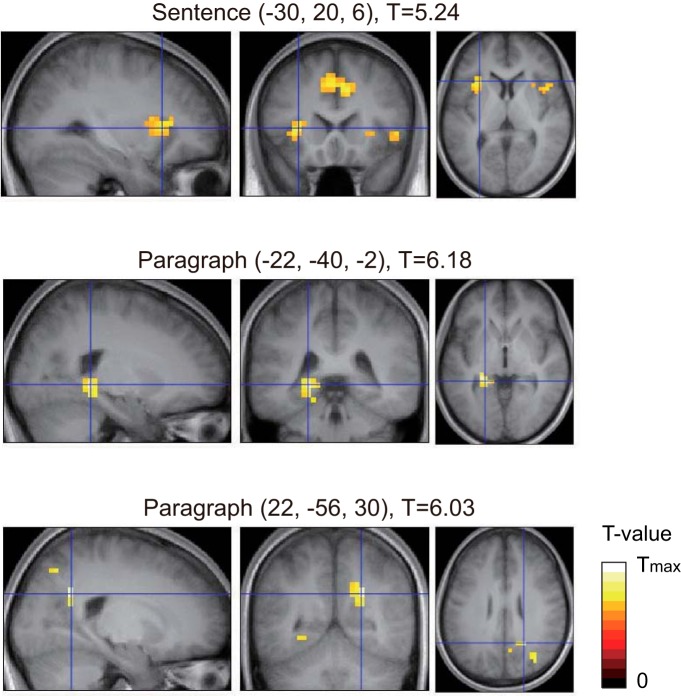
Brain regions showing a significant decrease in BOLD activity during reading of texts that were highly correlated with the content reports subsequently written by the participants. The results of sentence-length text correlation are shown on the top and in the middle, and those relevant to paragraph-length text correlation are shown on the bottom. There were no significant regions showing positive subsequent memory effect (i.e., BOLD activity increasing during reading of texts that were highly correlated with the content reports).

BOLD activities during text reading were analyzed by the time series of text correlations calculated with sentence-length and paragraph-length text comparisons between the text read and the content report. The results show no regions with a positive correlation between BOLD responses and the text correlation (i.e., no regions in which BOLD activity increased on reading the texts that were highly correlated with the content reports). In contrast, multiple regions showed significant negative correlation between the BOLD response and the text correlations (i.e., BOLD decreases during reading with high correlations between content reports and text being read; Fig. 3; Table 1). The sentence-length NSME was found in bilateral insula (BA13), right inferior frontal gyrus (BA47), and anterior cingulate gyrus (BA32). The paragraph-length NSME was observed in the left hippocampus/parahippocampal gyrus, the right precuneus/posterior cingulate gyrus (BA31), and the right intraparietal sulcus (BA7).

**Table 1.** Brain regions showing a significant decrease in BOLD activity correlated with sentence-length and paragraph-length text correlations

Anatomical region	MNI coordinates (mm)			*t* value
	x	y	z	
Sentence length (NSME)				
R-insula (BA13)	50	0	-10	7.09
R-inferior frontal gyrus (BA47)	46	16	2	4.61
R-cingulate gyrus (BA32)	10	24	38	5.90
L-cingulate gyrus (BA32)	-10	24	42	5.22
L-insula (BA13)	-30	20	6	5.24
Paragraph length (NSME)				
L-hippocampus/parahippocampal gyrus (BA36)	-22	-40	-2	6.18
R-precuneus/cingulate gyrus (BA31)	22	-56	30	6.03
R-intraparietal sulcus (BA7)	30	-72	38	4.76

*p* < 0.001 (uncorrected) with a cluster-wise FDR of *p* < 0.05.

## Discussion

We found that literature reading produced a positive relationship of fixation-related EEG and negative relationship of BOLD activity to subsequent memory as measured by semantic correlation between the text read and the content reports subsequently written by the participants. The following sections discuss the potential importance of these results for the understanding of neural dynamics of memory encoding during literature reading.

### Positive relationship between fixation-related EEG and subsequent memory

In the results, the sentence-length and paragraph-length effects were differently associated with FRPs and fixation-related EEG θ, respectively (Fig. 2). First, the sentence-length effects were positively associated with N1-P2 and P3 components (Fig. 2*A*). There are no reports, to our knowledge, on the relationship between FRPs and subsequent memory, although many researchers have reported increased P3 ERP components during successful encoding (Fernández et al., 1999; Klimesch et al., 2000). The current results agree with these data. On the other hand, the current results of the N1-P2 component does not simply agree with previous results; the current results appeared similar to the reading-related ERP components within 100–200 ms (mainly N1, but also the P2 component), which is thought to be associated with lexical word access (Sereno et al., 1998; Sereno and Rayner, 2003). However, topographic maps of these results, including both increases and decreases of the reading-related ERP amplitudes in multiple regions, are not directly associated with the current data, except for the left-hemisphere dominance. In addition to FRP, N1 ERP has been extensively investigated and is thought to be associated with visual discrimination process (for review, see Luck et al., 2000). This suggests that the current result of decreased N1 amplitude indicates a decrease in the visual discrimination process. In summary, the interpretations of the P3 and N1-P2 components could be combined as follows. The decreased lexical access and/or decreased visual discrimination interpreted by the N1-P2 can be explained by many factors, while factors related to “low mnemonic load,” rather than factors related to resting, would agree with the successful encoding interpreted by the P3. Furthermore, the low mnemonic load was speculated to be associated with “known contents” (or a good correspondence should be addressed to preexisting knowledge during reading) rather than “poor contents,” because enough semantic contents in the read text were required to detect successful encoding by text correlation in the current study. It should be noted that the current results did not include either N1 FRP at the occipital region, which was shown to be associated with reading (Henderson et al., 2013), or N400 ERP (Kutas and Hillyard, 1980; Kutas and Federmeier, 2000) and N400 FRP (Dimigen et al., 2011), which are shown to be associated with text comprehension.

Second, the paragraph-length effect was associated with increased EEG θ (Fig. 2*D*,*E*). There are a number of reports showing increased EEG θ during successful encoding (Klimesch et al., 1996; Weiss and Rappelsberger, 2000; Sederberg et al., 2003; Summerfield and Mangels, 2005; Osipova et al., 2006). The current results agree with these data, except for the topographic maps of the SME; the current results appeared in fronto-temporal and occipital regions, while previous reports differently appeared in frontal (Summerfield and Mangels, 2005; White et al., 2013), central (Klimesch et al., 2001), or temporal regions (Hanslmayr et al., 2011; for review, see Hsieh and Ranganath, 2014). Besides the successful encoding, the current results could be also associated with a more extensive mnemonic processes, such as linguistic comprehension associated with left fronto-temporal θ increases (Hagoort et al., 2004), or a higher load of cognitive control associated with left fronto-temporal θ increases (Sauseng et al., 2010).

### Negative relationship between BOLD activity and subsequent memory

Increases (Brewer, 1998; Wagner, 1998) as well as decreases (Otten and Rugg, 2001; Wagner and Davachi, 2001; Daselaar et al., 2004; de Chastelaine and Rugg, 2014) in BOLD activity have both been reported during successful encoding (for review, see Kim, 2011). Our results failed to identify regions showing positive relationships between BOLD signals and subsequent memory, while multiple regions showed negative correlations with subsequent memory (i.e., NSME), with the bilateral insula, right inferior frontal gyrus and anterior cingulate gyrus showing the sentence-length effect and the left hippocampus/parahippocampal gyrus, the right precuneus/cingulate gyrus, and the right inferior frontal gyrus showing the paragraph-length effect (Fig. 3; Table 1).

For the interpretation of the current results, the overlaps of the resultant regions with SME/NSME regions and other functional networks in previous reports were computed (Table 2). Surprisingly, the current results were found to be not well correlated with the regions showing NSME in a previous report (Kim, 2011), suggesting that the regions showing negative relationships to subsequent memory do not simply reflect successful memory encoding. The regions showing a sentence-length effect are primarily associated with the anterior salience network (Menon and Uddin, 2010), which functions to identify the most relevant among several internal and extra-personal stimuli to guide behavior. In contrast, the regions showing a paragraph-length effect largely overlapped with the default mode network (DMN; Raichle et al., 2001), which is known to be activated during relaxed non-task states and self-oriented cognition, such as mind wandering or autobiographical memory retrieval, and is deactivated during performing cognitive tasks (for review, see Buckner et al., 2008; Raichle, 2015).

**Table 2.** Volume overlap ratio of the NSME regions in this study with previously reported functional networks

	SME/NSME networks (Kim, 2011)		Functional networks (Shirer et al., 2012)					
Anatomical region	SME	NSME	ASN	dDMN	vDMN	PN	LECN	VSN
Sentence length NSME								
Cingulate gyrus			0.92^*^	0.15			0.03	
L-insula			0.40^*^					
R-insula/inferior frontal gyrus	0.41^*^		0.37^*^					0.05
Paragraph-length NSME								
R-precuneus/cingulate gyrus		0.18^*^		0.41^*^	0.20	0.37^*^		0.04
L-hippocampus/parahippocampal gyrus				0.01	0.04			
R-intraparietal sulcus					0.35^*^	0.11	0.51^*^	0.37^*^

SME/NSME networks were defined by multiple spheres of which locations and volumes were given by SME (verbal associative subgroup) and NSME (verbal item subgroup; Table 3 and 6 in Kim, 2011, respectively). Functional ROIs reported by Shirer et al. (2012) were analyzed. 6/14 functional networks showing significant overlap are listed in the table. Volume overlap was defined by (overlapped voxels)/(#voxels in the ROI). Each functional network was inflated ±1 voxel (3 mm) to give stable overlap. * indicates significance at the level of *p* < 0.05 with FDR correction (*q* < 0.05), with a null hypothesis of “volume overlap ratio between ROIs and functional regions is equal to the volume ratio of the region to the domain (voxels included in every network)”. ASN: anterior salience network; dDMN and vDMN: dorsal and ventral DMNs; PN: precuneus network; LECN: left-executive control network; VSN: visuospatial network.

The result of sentence-length NSME in the salience network could superficially produce a contradictive interpretation, i.e., successful encoding was associated with decreased attention to the text. However, this can be solved by considering the details of the decreased attention as follows. The salience network was thought to be continuously activated during reading, while its activity is supposed to be relatively decreased during the reading of texts characterized by low mnemonic load, such as “known texts,” as illustrated by the EEG sentence-length effect. Cognitive factors related to resting, e.g., low wakefulness or low interest to the text, were also associated with decreased attention to the text. However, these effects, of which time course was supposed to be longer than the time course of individual sentence reading, were expected to be regressed out from the sentence-length effects in the current multiple regressions analysis, and that would be dominant in the paragraph-length effect. The texts characterized by low mnemonic load were supposed to be easily memorized, thus the sentence-length NSME in the salience network is explained

The result of paragraph-length NSME in the DMN was explained by the suppression of task-irrelevant internal thoughts (Anticevic et al., 2012) and the effective allocation of cortical resources (Hasson et al., 2007). In contrast to the salience network, the DMN was thought to be continuously deactivated during reading, while the DMN could be more strongly deactivated during higher efforts of reading when task-irrelevant internal thoughts were more strongly suppressed. During such periods, the encoding performance was also supposed to be better. As a result, the paragraph-length NSME in the DMN would appear. As discussed above, with the consideration of the time course of the task-irrelevant internal thoughts, this effect would likely appear as a paragraph-length effect, rather than a sentence-length effect.

The results of NSMEs in both the salience network and the DMN seemingly contradict that these networks are usually known to have opposite activation patterns. However, this is simply solved by considering the differences of the time courses of the detected BOLD signals in these networks. In the current essays used, the average paragraph-length was two-and-a-half-times longer than the average sentence-length (See EEG Methods/Stimuli); accordingly, the time course of the paragraph-length regressors was sufficiently longer than the time course of the sentence-length regressor. Thus, it was thought that the NSMEs in the salience network and the DMN were not contradictory, but instead reflected differences in cognitive aspects in different time courses; the former reflected the reading of individual sentences characterized by low mnemonic load and the latter reflected the cognitive states related to the suppression of task-irreverent thoughts.

The hippocampus usually showed a positive SME, but our results showed an NSME for the left hippocampus/parahippocampal gyrus. This failure to detect increased BOLD activity during successful encoding may be explained by the long-lasting reading (∼10 min) in the current task, in which the regions associated with subsequent memory were continuously activated. A relatively small fluctuation dependent on subsequent memory may be obscured by a more general pattern of task-related deactivation, as pointed out by Yarkoni et al. (2008). Recently, Baldassano et al. (2017) demonstrated that the hippocampus was specifically activated at the end of an event, while the average hippocampal activity was decreased during the event. This may also explain the current result.

### Relationship between fixation-related EEG and BOLD activity

Simultaneous fMRI-EEG measurements have demonstrated an inverse correlation between frontal EEG θ and BOLD activity in the DMN during resting (Scheeringa et al., 2008), mental arithmetic (Mizuhara et al., 2004), and memory encoding (Sato et al., 2010; White et al., 2013). The current results for the paragraph-length comparison showed a negative relationship with fronto-temporal EEG θ and a positive relationship with BOLD activity in the DMN, which is in agreement with these previous reports.

On the other hand, combined ERP-fMRI studies have demonstrated a positive correlation between P3a components (an earlier subcomponent of P3) and BOLD activities in frontal areas and the insula during oddball tasks (Bledowski et al., 2004), and in the anterior cingulate during spatial attention tasks (Bengson et al., 2015). The current results of the sentence-length comparison, showing a positive relationship with N1-P2 and P3 FRP components and a negative relationship with BOLD activity in the salience network, does not agree with these previous reports. This might be explained by differences in the cognitive tasks; the current task of natural reading required more complicated cognitive processes than the tasks described in the previous reports. Another reason is thought that ERPs originating from phase resetting might not induce major changes in local brain metabolism, as pointed out by Debener et al. (2006).

### Text correlation as an index of subsequent memory

There are different levels of text comprehension; textbase comprehension is the encoding of the meaning of the text and situation model construction is further supplemented by preexisting knowledge needed for coherent understanding (Kintsch, 1994). Free-recall tasks have been typically used for the evaluation of textbase comprehension; however, the text correlation used in the current study was thought to measure subsequent memory associated with both the textbase and situation model. One reason for this was that the text read was long enough (∼8 and 4 thousand words, i.e., ∼30 and 15 min of reading, in EEG and fMRI experiments, respectively) to allow memorization of phrases in the text; however, semantic context and preexisting knowledge were still available during reading as effective guides for encoding. This may lead participants to use a strategy that falls within the situation model for the encoding of text. This was partially supported by the results of content reports, each of which consisted of a small number of words (∼10% of the text read), but included abstract representations of the text read. On the other hand, the possible use of preexisting knowledge during reading may produce a problematic variation in encoding strategies. Unfortunately, the current study cannot rule out this possibility; however, behavioral results showing no explicit outliers in reading time and the word number of content reports suggests a relatively small variation in participant encoding strategies.

In summary, our results demonstrated (1) the availability of semantic correlation of text, based on a recent natural language processing procedure, to detect brain activities related to context-dependent memory; (2) the positive relationship of FRP and fixation-related EEG θ and negative relationship of BOLD activity with subsequent memory; and (3) the different time courses of memory-related activities in the salience network and the DMN during reading, in which the sentence-length encoding was associated with salience network deactivation (thought to reflect the reading of sentences characterized by low mnemonic load), and the paragraph-length encoding was associated with the DMN deactivation (thought to reflect the suppression of task-irreverent thoughts during reading). It has been suggested that context-dependent memory encoding during natural reading of literature requires large-scale network deactivation that might reflect the coordination of a range of voluntary processes during reading.

## Synthesis

Reviewing Editor: Bradley Postle, University of Wisconsin Decisions are customarily a result of the Reviewing Editor and the peer reviewers coming together and discussing their recommendations until a consensus is reached. When revisions are invited, a fact-based synthesis statement explaining their decision and outlining what is needed to prepare a revision will be listed below. The following reviewer(s) agreed to reveal their identity: Ashley Lewis, Sepideh Sadaghiani.

Please note that both reviewers raise questions/concerns about the approach of comparing across rank-ordered subjects from the fMRI and EEG groups. Although both suggest in their reviews that this be removed from the manuscript (see below), after consultation between the three of us an alternative option arose that you might consider: removing all but a sentence of reference from the main body of the paper, and including this as an exploratory, “hypothesis-generating” analysis that is described in an addendum.

Here is the full text of the two reviews:

Reviewer #1

Summary:

This study applied state of the art natural language processing procedures for summarizing the semantic content of linguistic materials in a contextually rich narrative-reading and subsequent memory paradigm. These metrics were used to quantify the semantic content of both narrative texts read by participants, and of subsequently produced summary reports of the contents of those texts. Similarity between the semantic content of the reports themselves and the texts read was computed as an indication of subsequent memory. These measures were then correlated with EEG theta oscillations and BOLD activation measured at each content word read in the narrative texts during encoding in order to investigate how neural signatures were related to this measure of subsequent memory. The study demonstrated a positive relationship between subsequent memory and theta power, and a negative relationship between subsequent memory and BOLD activation in a network of regions that constitute parts of the default mode network (and one relationship with regions forming part of the salience network). A relationship was also demonstrated (although only a marginal result) between the positive EEG relationship with subsequent memory and the negative BOLD relationship with subsequent memory. The authors conclude that memory encoding during narrative comprehension requires active suppression of a range of voluntary processes related to self-oriented cognition and the default mode network.

This study offers a potentially exciting and novel approach to investigating neural activity related to the semantic contents of subsequent memory effects in more naturalistic text reading contexts. There are however a number of problematic aspects of the paper in its current form. First, there are a number of methodological details that are either missing or inappropriate and need to be fixed. Second, there are a number of issues with how the results are presented and interpreted. Third, it's not clear that the authors have done enough to establish that their measure of subsequent memory is comparable to those used in typical subsequent memory paradigms. I provide more detailed comments regarding each of these points below, followed by some miscellaneous minor comments.

Methodology:

Pg.4/ln28-29: “At the beginning of each reading session, the eye movement system was calibrated with a 9-point grid.” This is the first mention that the study would additionally involve eye tracking. I would introduce this earlier by indicating that the study would be combined EEG and eye tracking already near the end of the introduction. This is a less common method of stimulus presentation for EEG and fMRI studies, and so it should be pointed out early on and the technique's validity should be established by citing some literature supporting its use. Specifically, the ability to deal with eye movement artifacts in the EEG needs to be established.

Pg5/ln23:28: The description of how independent components analysis was used to correct the data for ocular artifacts needs to provide more details of precisely what was done. Since this study uses a combination of EEG and eye tracking where the eyes are expected to move across the text being read it needs to be established whether or not eye movement artifacts were sufficiently dealt with. First, it is not clear that one can obtain a reasonable ICA decomposition based on only 170 ms of data (-120 to 50 ms relative to fixation onset). Please provide more information about how much data (e.g., how many trials, time points per trial, number of electrodes) was entered into the ICA decomposition, and how it was established that stable ICA components were obtained. See for example Artoni et al., 2014: RELICA: A method for estimating the reliability of independent components. Second, the authors state “... and the same separation matrix was applied to the original data.” If the ICA was performed on a subset of the data not used in the eventual analyses, the authors should please clearly state that and provide any details of additional filtering and/or re-referencing etc. that may have been employed to optimize the ICA decomposition. Third, the authors state that “Independent components highly correlated to either horizontal, vertical, or radial EOGs (correlation coefficient > 0.15) were discarded ...”. Please specify what kind of correlation was performed, e.g., was it a correlation of the entire time-course across trials or a correlation across time of the average time-course. It would also be useful to have some indication of what the data look like before and after correction of eye movement artifacts. As it stands Figure 2 presents only regression values, so it's difficult to assess whether the ICA approach employed adequately dealt with the EOG artifacts in the data.

Pg.5/ln29-30: “... and down-sampled to 250 Hz to match with eye-movement data.” The authors should please specify precisely how the EEG data were co-registered with the eye movement data. If this was achieved with a common time-stamp for instance then down-sampling can be problematic.

Pg.6/ln26-27: “Third, three types of correlation were calculated using sentence-length, paragraph-length, or the entire text correlation.” What was taken to constitute sentence-length and paragraph-length, and were these always approximately equivalent? Would the output of these correlation analyses be expected to be sensitive to the amount of information entered, and if so then how was this controlled for when working with sentences or paragraphs of different lengths? The authors should please provide a clearer description of this aspect of the text analysis. The entire description of what precisely was done for the text analysis is not very clear, and it would be useful if the authors could clearly outline the specific steps (with a step-by-step list for instance) involved in getting from the text input to the sematic vectors and how these are correlated between the content reports and the texts read.

Pg.6/ln24-26: “The feature dimension (100) was determined to maximize the difference between the text read-content report correlation and the surrogated text correlation.” This is the first time a ‘surrogated text correlation’ was mentioned. The authors should please introduce this concept earlier and describe what it is, how it is computed, and what it's purpose is.

Pg.9/ln18-28: It's unclear whether the fMRI experiment also used eye tracking during reading, and if so how this was co-registered with the fMRI data. This has implications for how the semantic vectors from the content reports were correlated with those for the text read. Please provide a more detailed account of how this was done.

Pg.17/24-26: “The spatial pattern of the regression coefficients that was not focused in the frontal region and continuously in time showed that the influence of ocular artifact residuals on the current results was negligible.” This is simply not true. The spatial distribution of the regression coefficients shows where the effect is largest, but the measures at these regions can very well be contaminated with residual ocular artifacts. The maxima of the ocular artifacts would certainly be located near the front of the head, but in case such artifacts are present (and the topographies presented do not speak to that because they present only correlation coefficients) they typically have an influence on all electrodes.

Presentation/interpretation of results:

Pg.1/ln16: “... with the theta/alpha timing suppression hypothesis ...” There is no such hypothesis as far as I am aware. Here and in the discussion section the authors conflate the theta-gamma code (Lisman & Jensen, 2013) and the inhibition timing hypothesis (Klimesch et al., 2007) or gating by inhibition hypothesis (Jensen & Mazaheri, 2010). The latter two are referring specifically to the role of alpha oscillations in cortical inhibition. The former refers specifically to the ordering of information within working memory based on gamma oscillations nested at different phases within a theta cycle. This theta-gamma code does not have anything to do with cortical inhibition in the way the authors want to claim, and so their claims about an inhibitory role for theta oscillations needs to garner support from elsewhere, or alternatively be argued for on the basis of the data presented in this paper.

Pg.2/ln9-12: “In contrast, the activation of certain brain regions during ...” It's not enough to just point out that some brain regions show a reverse effect for subsequent forgetting. Please be specific throughout the manuscript about which brain regions are expected to show greater BOLD activation for a subsequent memory effect (SME) and which brain regions are expected to show greater BOLD activation for a subsequent forgetting effect (NSME), and also whether or not and how these regions overlap.

Pg.2/ln17-20: “... EEG theta and EEG at other frequency bands, such as alpha-band (8-10 Hz) and beta-band (13-15 Hz) EEG, were also shown to associate with either SME or NSME ...” First, the beta range is wider than 13-15 Hz so please could the authors clarify whether this is a typographical error? Second, the authors should please be more specific here. Which effects (SME or NSME) have been related to which oscillatory activity and under which circumstances in relation to which cognitive or mnemonic function?

Pg.2/ln20-23: “... scalp EEG theta was positively or negatively correlated with the blood-oxygenation-level dependent (BOLD) activity ...” Please specify which of these options it actually is. If it's both positively and negatively correlated, then you need to specify how this could be the case, i.e., is it positively correlated for some brain regions and negatively correlated for others, or alternatively is the positive correlation observed under some task demands and the negative one under other task demands, or alternatively is the positive correlation present at different times than the negative correlation? Please be more specific.

Pg.11/ln11: “... showed a significant increase in theta-band EEG power ...” Throughout the results and discussion section the authors describe an increase or a decrease in theta power or in BOLD activation but based on what has been reported to have been tested it was never established whether theta power or BOLD exhibit increases or decreases. What is reported are regression coefficients, and this only tells one about the relationship between these neural measures and the metric with which it's being related. To claim an increase or decrease the authors would need to show that this is what's present in the data, and that that is then related to the semantic correlations in a particular way. The authors should please either establish this by presenting the actual data and an analysis showing such increases and decreases, or alternatively be more careful with their descriptions of their results by referring instead to positive and negative relationships. This becomes particularly important when they wish to make claims about whether there is an increase or decrease in BOLD or in theta power related to the subsequent memory effect or the subsequent forgetting effect, because what's demonstrated does not currently provide information about whether activation in a particular region is related to worse memory performance, or instead deactivation in that region is related to better memory performance. This issue is pervasive throughout results, discussion, and abstract sections.

Pg.11/ln13: Here and in a number of other places the authors provide statistical output for maximal effects, but it's unclear whether what's reported are corrected or uncorrected values (this is not the reporting format one would typically see for corrected values based on the cluster-based permutation approach). I suspect these are uncorrected values, in which case I don't see where the corrected statistical output is reported. Could the authors please clarify and if necessary additionally report statistical results corrected for multiple comparisons.

Pg.11/ln22-23: “This result suggested that the increase in EEG theta during successful encoding did not result from the contamination by ocular artifacts.” The authors are willing to interpret marginal effects later in the manuscript as indicating relationships they wish to discuss between the EEG and fMRI data for instance. They should then apply the same standards here and admit that there is a marginal effect for fixation duration for both sentence-length (0.08) and paragraph-length (0.07) correlations. This suggests that fixation duration may actually be a confounding factor for the observed theta power effects (theta power may simply be higher when a word is fixated longer for instance). I think the authors need to address this issue by for instance running their regression models with fixation duration as an additional regressor (similar to what was done for the fMRI analysis) to see whether this indeed changes the outcome.

Pg15/ln12-30: This entire section should be removed. Nowhere in the present study has it been demonstrated that there is coupling between increased EEG theta oscillations and decreased BOLD. At best the authors have a marginal effect suggesting that increased EEG theta and decreased BOLD are related to similar correlations between the semantic content of the content reports and the semantic content of texts read. To establish coupling between these measures one would have to demonstrate that there is a direct relationship between increased EEG theta and decreased BOLD (i.e., between the brain signals themselves). Ideally one would also like to show that this coupling is then related to the subsequent memory effects quantified as described above but establishing this relationship independently for each neural index is not sufficient to indicate coupling between those measures. The authors state that “The results of ROI analysis showing the dependence of memory performance on the sentence-length effect, but not the paragraph-length effect (Figure 4C), supported this coupling.” This line of argument doesn't make any sense and certainly doesn't speak to the coupling between the EEG and the BOLD measures.

Pg.16/ln20: “... (2) hierarchically organized suppression mechanism ...” As far as I can see this is the first mention of a hierarchically organized suppression mechanism (apart from in the abstract). No argument has been provided for how this has been established as hierarchical by the data presented, and I can't find any attempt by the authors to try to justify this claim.

Figures 2 and 3: It looks very much like the data presented in these figures are masked. If so please could the authors provide a description of what they are masked by (e.g., a statistical mask? corrected for multiple comparisons?). It would be very useful for the time-frequency data to also provide a figure illustrating the grand-average power values that correspond to the detected effects. This would allow one to assess whether power exhibits an increase or a decrease, and how successful ocular correction was.

Subsequent memory:

Pg.3/ln17-18: “These studies were important in that they supported the plausibility of using intermediate semantic features in the evaluation of subsequent memory during reading.” I understand how these studies support the plausibility of using intermediate semantic features to investigate brain activity related to semantics, but I don't follow how this offers any support for using this approach in subsequent memory. Could the authors please expand on the logic of this argument.

Pg.4/ln32-Pg.5/ln1: “During this procedure, the interval between essay encoding and retrieval was of approximately 30 min, and no explicit opportunities for rehearsing the essays read were included.” While it may be the case that no explicit rehearsal was encouraged, not having an explicit task in the interval between encoding and subsequent memory test makes it difficult to know precisely what participants were doing in this interval. Some may have been rehearsing, and others not, but there are a whole range of possibilities, making it difficult to relate the performance on the subsequent memory task specifically to encoding during narrative reading (it could be related to anything that happened in this interval between encoding and subsequent memory test). This is a serious flaw in the study's design, and it is a problem for both the EEG and the fMRI experiments.

Pg.6/ln10-12: “The text read and the content reports subsequently written by the participants were individually translated into semantic vectors, and their correlation was used as an index for semantic text correlation.” I agree that this provides a measure of the degree of semantic overlap between the content reports and the text read based on this metric, but I think the authors need to do a bit more work explaining why they think this is an adequate measure of subsequent memory performance. For example, one could imagine that without comprehending the text at all a participant could write down a number of disjointed words that appeared in the text and still achieve a high score for degree of semantic overlap. This is an extreme example of course, but the point is that there needs to be more justification for why this measure is appropriate to capture subsequent memory performance, and possibly some discussion of whether or not this is intended to specifically capture cognitive function (i.e., do the authors intend the measure as capturing the reality of what the cognitive system is doing) related to subsequent memory.

Pg.8/ln27-29: “... wrote two content reports within 15 min following the order in which the essays were read in as much detail as possible.” It's all well and good to ask people to write a content report in as much detail as possible, but it's quite likely that there is a lot of variability between participants in how they would approach this task. This is something that is typically well controlled in standard subsequent memory paradigms (a recognition probe is presented for instance, which allows only a limited number of possible responses), but in this paradigm there are many factors that could be at play and could vary quite widely between or even within participants during the subsequent memory test. The authors should please provide some discussion of these points and justification for why this is not problematic for their study.

Pg.12/ln26-28: “We reported that increased EEG theta and decreased BOLD activity during literature reading predicted subsequent memory success measured by semantic correlation between the text read and the content reports subsequently written by the participants.” I think this claim is a little too strong. I would argue that the authors have shown that there is a relationship between EEG theta as well as BOLD and the measure of semantic overlap between content reports and the text read, but whether or not this is capturing subsequent memory in the way subsequent memory is standardly understood has not really been established in this study. More work would need to be done to establish the adequacy of the paradigm for capturing the standard subsequent memory effects before such a claim could be made.

Minor Comments:

Pg.1/ln7: “... were analyzed by comparing the results obtained with the reports subsequently written ...” It's unclear what ‘results obtained’ refers to. It's likely to refer back to neural signals measured with the techniques mentioned in the previous sentence, but please be more specific here.

Pg.3/ln1: “... have been evaluated by fMRI studies...” Narrative level context has been evaluated by EEG/MEG studies as well (see e.g., Brennan, 2016: Naturalistic Sentence Comprehension in the Brain) so it may be worth citing some of those here too.

Pg.3/ln9-10: “Recent techniques in natural-language processing, a quickly growing subfield of computer science, have been applied for quantifying semantic content in the subsequent memory.” Please provide some citations for this work specifically applied to subsequent memory.

Pg.4/ln3-8: It's customary to mention all exclusion criteria so I'm curious whether there were any more than handedness? Could the authors please add details about whether participants were excluded based for instance on neurological disorder, visual acuity, the use of psychotropic medications, etc. Please also mention whether and how participants were compensated for the experiment.

Pg.5/ln19-21: “... and re-referenced to linked earlobes ...” I think the authors mean to say ‘re-referenced to the average signal recorded at electrodes placed on the two earlobes’. A linked mastoid reference implies that the electrodes at each earlobe are physically connected during recording.

Pg.5/ln15-21: The authors should please report the impedance threshold used to ensure good contact between the recording electrodes and the scalp.

Pg.6/ln1-12: Here where intermediate semantic features are introduced I would like to see a better description of precisely what these intermediate semantic features are and what aspect of the texts they are designed to capture. In other words, what does it mean to have summarized a text or a word/sentence/paragraph based on intermediate semantic features and why is that useful for the current study.

Pg.7/ln9: “... data points now within 80 μV were ...” Not clear within 80 μV of what? I think the authors mean to say that a threshold of +/- 80 μV was used.

Pg.7/ln15-16: The baseline period is mentioned but it is not stated whether a baseline correction was employed. Please clarify.

Pg.7/ln29: 500 shuffled data sets is quite a low number to use for constructing the monte carlo distribution in the nonparametric clustering permutation test used to correct for multiple comparisons. It is unclear whether this will achieve a stable estimate of the permutation distribution. I would suggest that the authors try at least 1000 randomizations, more if possible.

Pg.8/ln25-26: It's not clear why the presentation of the texts was different in the fMRI experiment than in the EEG experiment. The authors should please provide an explanation for why this was the case.

Pg.10/ln14-19: This description does not fully specify how fMRI and EEG results were compared. What measure was used to assess the degree to which these results were related? Please specify this clearly here in the methods section. If a correlation approach was used then any conclusions drawn would have to be tentative because of the low number of data points (13 participants).

Pg.10/ln24-25: Here and throughout the description of the results numbers are presented to indicate how long it took to read texts or write content reports etc., which I assume indicate something like means and standard errors or standard deviations. There were however 2 or 4 texts read and summarized per participant, so it's not clear exactly what these numbers refer to (means of means?). Please specify this more clearly.

Pg.10/ln33: “The memory performance was calculated as ...” Please specify that the numbers here are based on an analysis excluding the two outliers in Figure 1B, otherwise the numbers don't correspond well to what's presented in the figure.

Pg.11/ln6: “After rejecting atypical saccades ...” Please describe (approximately) the criteria used for deciding whether or not a saccade was atypical.

Pg.11/ln14-18: “... averaged over time period ...” Please specify what this time period was for each.

Pg.11/ln32-33: “The sentence-length NSME were found in bilateral inferior frontal gyrus/insula ...” It's a little tricky to lump together the inferior frontal gyrus and the insula. These are two functionally and cytoarchitectonically distinct regions and are quite distant in fMRI space. Please justify or change.

Pg14/ln14-16: The authors' description of the default mode network is not quite accurate. Activity in this network is typically high when no particular task is being performed (e.g., under conditions of mind-wandering or self-oriented cognition) but shows decreased activation when participants are presented with a cognitive task. So, the network is actually active during relaxed non-task states, contrary to what the authors claim, and shows decreased activation when presented with a task.

Reviewer #2

I think the multi-modal design in combination with the semantic correlation approach has resulted in a valuable description of brain activity during memory encoding of natural reading. I have three major comments:

The participants in EEG and fMRI investigations were different groups, unfortunately. The authors try to directly link EEG and fMRI observations by ranking individuals in both groups, then matching pairs across groups by rank. Such pairing is not straightforward, and the resulting statistical cross-modal correlation analysis is very weak (t(11)=1.83, page 12). I suggest removing this analysis and any reference to cross-modal investigations of these non-overlapping subject groups.

Regarding the anatomical and functional interpretation of fMRI results, I do not think that the network of regions showing reduced activity for sentence-length memory is a DMN subcomponent. Judged by the anatomical description and the image provided it rather looks like the Cingulo-Opercular / Salience network. I suggest that the activation maps be overlaid on resting-state functional connectivity maps of the DMN and Cingulo-Opercular / Salience networks to clarify this point (volume overlap could also be quantified if in doubt). Resting state connectivity maps are available online from many groups, e.g. https://findlab.stanford.edu/functional_ROIs.html , as well as the Buckner group that the authors refer to in their description of the DMN.

In case the sentence-related effects are indeed in the CO/salience areas, the interpretation of reduced activity linked to better memory becomes tricky and must be carefully revised.

I am not sure I understood the fMRI ROI definition correctly specifically as it relates to avoiding circularity. Although the ROI is defined for each subject in a leave-one-out procedure, it sounds like the resulting peak was defined within a sphere that was taken from the entire subject group including the left-out subject (is that what is meant by “original result” on p. 10 lines 8-9)?
